# Motion Artifact Reduction in Wearable Photoplethysmography Based on Multi-Channel Sensors with Multiple Wavelengths

**DOI:** 10.3390/s20051493

**Published:** 2020-03-09

**Authors:** Jongshill Lee, Minseong Kim, Hoon-Ki Park, In Young Kim

**Affiliations:** 1 Department of Biomedical Engineering, Hanyang University, Seoul 04763, Korea; netlee@hanyang.ac.kr (J.L.); minseong5905@hanyang.ac.kr (M.K.); 2 Department of Family Medicine, Hanyang University, Seoul 04763, Korea

**Keywords:** photoplethysmography, motion artifact, independent component analysis, multi-wavelength

## Abstract

Photoplethysmography (PPG) is an easy and convenient method by which to measure heart rate (HR). However, PPG signals that optically measure volumetric changes in blood are not robust to motion artifacts. In this paper, we develop a PPG measuring system based on multi-channel sensors with multiple wavelengths and propose a motion artifact reduction algorithm using independent component analysis (ICA). We also propose a truncated singular value decomposition for 12-channel PPG signals, which contain direction and depth information measured using the developed multi-channel PPG measurement system. The performance of the proposed method is evaluated against the R-peaks of an electrocardiogram in terms of sensitivity (Se), positive predictive value (PPV), and failed detection rate (FDR). The experimental results show that Se, PPV, and FDR were 99%, 99.55%, and 0.45% for walking, 96.28%, 99.24%, and 0.77% for fast walking, and 82.49%, 99.83%, and 0.17% for running, respectively. The evaluation shows that the proposed method is effective in reducing errors in HR estimation from PPG signals with motion artifacts in intensive motion situations such as fast walking and running.

## 1. Introduction

Photoplethysmography (PPG) is an optical method used to detect volume changes in blood in the peripheral circulation. PPG can determine these volume changes from the surface of the skin, and is a low-cost and noninvasive method. This technique provides useful information related to cardiovascular systems such as heart rate, oxygen saturation, blood pressure [1], and cardiac output [2,3], and is also used to determine stress levels by analyzing the response of the autonomic nervous system based on pulse rate variability (PRV) [4]. 

Recently, wearable PPG sensors have attracted attention because they can continuously measure and monitor heart rate (HR), and numerous devices in the form of bands or watches (e.g., Apple watch, Fitbit, and Samsung Gear) are being used to monitor instantaneous heart rate using PPG. While these PPG-based devices have the advantages of being lightweight, portable, and easy to use, the distortion of the signal due to motion artifacts in the PPG signal is a challenge to overcome. Currently, these devices are only used for general wellness purposes because they are accurate only in limited conditions such as resting or walking slowly.

PPG uses a sensor composed of light-emitting and light-receiving elements. When light is irradiated to the body tissue by the light-emitting element, it is transmitted, reflected, and scattered by the Beer–Lambert law in the tissues, blood vessels, and blood of the body and detected by the light-receiving element [5,6]. In general, a green, red, or infrared light-emitting diode (LED) is used as a light-emitting unit, and a photo diode (PD) is used for a light-receiving unit [7]. LEDs used for PPG measurements generally have a wavelength of 400–1000 nm. Short wavelengths do not reveal much cardiac activity and blood vessel information due to low skin penetration depths, but are less affected by motion artifacts due to the shorter light path. In the case of long wavelengths, the penetration depth of the skin is deep, which can clearly indicate activity of the heart and blood vessels such as the dicrotic notch, but these are affected by motion artifacts because of the long light path [8].

The normal frequency range for PPG signals is 0.5 to 5 Hz, while for motion artifacts it is 0.01 to 10 Hz [9,10,11]. Therefore, it is not easy to obtain a clean signal by applying a general filter to a PPG signal contaminated by motion artifacts. In order to solve this problem, adaptive filters or moving average filters are commonly used in the industrial field. However, satisfactory performance in removing or reducing motion artifacts has not yet been achieved, and various signal processing methods for reducing motion artifacts of PPG signals have been proposed. 

Poh M.-Z. et al. developed an earlobe-wearable PPG measuring device and presented a method of removing motion artifacts by applying adaptive noise cancellation (ANC) using an accelerometer [12]. The correlation between the heart rate calculated via electrocardiogram (ECG) and the heart rate measured via PPG was shown as a performance evaluation. The results showed a correlation coefficient of 0.75 (*p* < 0.001) when ANC was applied while running. Due to the size of the earlobe-wearable measurement system, it is difficult to apply in everyday life and it may be inconvenient when measuring because the attachment method uses neodymium magnets. In addition, the ANC method requires an additional sensor, such as an accelerometer, because it must provide a reference signal for motion artifacts. 

In a recent study, Zhang Y. et al. proposed the use of optical signals rather than accelerometers as the motion reference for the cancellation of motion artifacts [13]. The proposed framework uses the infrared (IR) PPG signal as the motion reference and the green PPG signal for HR estimation. This approach helps to reduce burden on additional hardware such as accelerometers and the computational complexity. 

Reddy K. A. et al. proposed the CFSA (Cycle-by-cycle Fourier Series Analysis) method using Fourier series analysis for each cycle using the autocorrelation of PPG signals [14]. The results show that randomly applied Gaussian noise is removed. However, due to the limitation that CFSA can only be applied to periodic signals, it is difficult to apply to situations where loss of periodicity occurs due to distortion caused by motion artifacts during the actual PPG measurement. 

In order to improve the performance of the noise reduction algorithm, methods using a multi-channel PPG system have been proposed. Warren K. et al. measured six-channel PPG signals at the forehead using six red and infrared LEDs [15]. They proposed an algorithm that selects the channel with the least influence of noise by quantifying the amount of motion artifacts for each channel during exercise. As a result, it was possible to automatically select an accurate channel from the measured multi-channel PPG signals. However, since the algorithm does not include noise reduction, and selects the signal with the lowest noise level from the measured signals, it is difficult to apply when motion artifacts exist in all channels. 

It is necessary to design a filter suitable for multiple channels, and various algorithms such as independent component analysis (ICA), principal component analysis (PCA), and singular value decomposition (SVD) have been actively studied [9,16,17,18,19]. PCA is used to find an orthogonal linear transformation that maximizes the variance of variables, whereas ICA is used to find the linear transformation of the basis vectors that are statistically independent and non-Gaussian. Unlike PCA, the major feature of ICA is that the basis vectors are neither orthogonal nor ranked in order. The PPG signal and the motion artifacts are independent components of the detected signal, so ICA or PCA can be used to separate the cleaned PPG signal from these artifacts. However, in most studies, the number of PPG channels used for verifying a multi-channel signal processing algorithm is limited, and the relationship between the multi-channel signal and the algorithm is not clear. 

In this study, we develop a multi-channel PPG measurement to consider the effects of motion artifacts on the direction of the sensor module and the change of penetration depth in the skin according to the wavelength. Further, we propose a multi-channel motion artifact reduction algorithm based on the signals obtained through this system. Using a multi-channel PPG system, 12-channel PPG signals for three wavelengths are acquired in four directions (up, down, left, and right). We present a method by which to reduce motion artifacts through applying ICA and a truncated SVD to 12 channels of PPG signals. We extract the independent components using an ICA of three channels of PPG signals measured in each module, then select the most pulsatile components. Using PCA, the statistical method by which the basis vectors are ranked in order, we can obtain the cleaned PPG signal. PCA can be implemented with powerful, robust techniques such as singular value decomposition (SVD) [20]. Ultimately, we implement PCA with SVD.

## 2. Materials and Methods

Our research focuses on reducing motion artifacts using multi-channel PPGs. In this section, we introduce our in-house-built wearable multi-channel PPG measurement system and describe the proposed motion artifact reduction algorithm using a multi-sensor module. We also describe in detail the experimental protocol and data acquisition using the measurement system.

### 2.1. Wearable Multi-Channel PPG System

**Figure 1:**
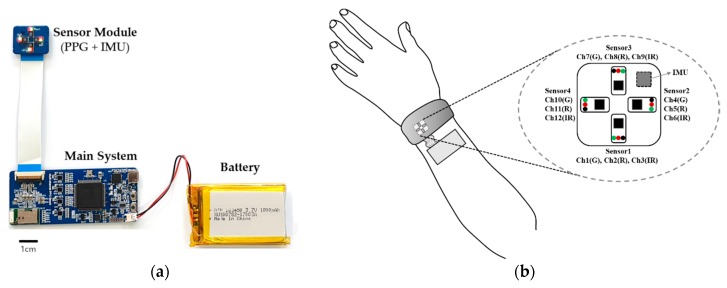
In-house-built wearable multi-channel PPG acquisition system: (**a**) wearable PPG hardware system consisting of a main system, sensor module (PPG and IMU), and battery; (**b**) the direction and wavelength (G: green; R: red; IR: infrared) of the sensor for each channel when the wearable system is worn on the wrist.

We developed a wearable multi-channel PPG system consisting of the main system, inertial measurement units (IMUs), and PPG sensors, as shown in Figure 1a. The sensor module connected to the main system has sensors arranged in four directions perpendicular to its center, with each sensor consisting of a green, red, and infrared LED and one PD (Figure 1b). In addition, the sensor module includes a nine-axis IMU to detect movement.

**Figure 2:**
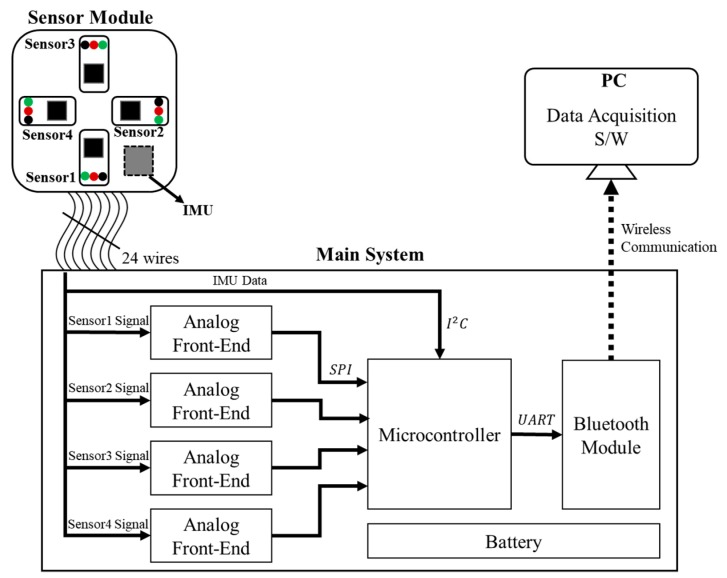
System diagram of the wearable multi-channel PPG acquisition system including PC application software (S/W). The solid line indicates a wired connection (USART (Universal Asynchronous Receiver/Transmitter), SPI (Serial Peripheral Interface) and I2C (Inter-Integrated Circuit)) while the dashed line indicates a wireless connection.

The system architecture of the in-house-built wearable multi-channel PPG measurement system is shown in Figure 2. The main system consists of an ARM Cortex TM-M4-based microcontroller (STM32F407VGT, STMicroelectronics, Geneva, Swiss), an analog front-end (AFE4900, Texas Instruments, Dallas, TX, USA), and a Bluetooth module (PAN1321i, Panasonic, Osaka, Japan). The sensor module was designed and implemented using four SFH7050 sensors (OSRAM, Munich, Germany) and one motion sensor (MPU9250, InvenSense, San Jose, CA, USA). SFH7050 is a sensor for heart rate monitoring or oximetry. It is an integrated sensor that contains three LEDs (green, red, and IR) of different wavelengths.

The readings of the PPG sensor are acquired via the analog front-end under the control of the microcontroller, then the amplified digital signals are sent to the microcontroller using serial peripheral interface (SPI) communication. Motion sensor data are transmitted to the microcontroller via I2C communication. The main system includes a Bluetooth module so that the data can be transferred to a PC via wireless communication. Additionally, we have implemented in-house data acquisition software for wireless transmission and storage to the PC.

The PPG signal of each channel was acquired at a 100-Hz sampling rate with a 24-bit high resolution, while the three-axis acceleration data were acquired at 16 bits and 100 Hz. In this paper, we use the vector sum magnitude of three-axis acceleration to determine the degree of motion.

### 2.2. Data Acquisition

The subjects were seven healthy males and one female without cardiovascular disease between the ages of 20 and 30 years (mean age: 27.1 years). This study was approved by Hanyang University IRB (IRB Approved no. HYI-17-048-4) and informed consent was received from all subjects before the experiment. 

Since ECG is characterized by robustness to motion artifacts, most studies use ECG as the ground truth [21,22]. Therefore, in this study, the experiment was conducted using the polar chest strap electrode with high reliability in ECG measurement. In addition, the electrode was wetted with water before the experiment to minimize contact noise between the skin and the electrode. The R-peak could be easily extracted from the measured ECG through the Pan–Tompkins algorithm.

**Figure 3:**
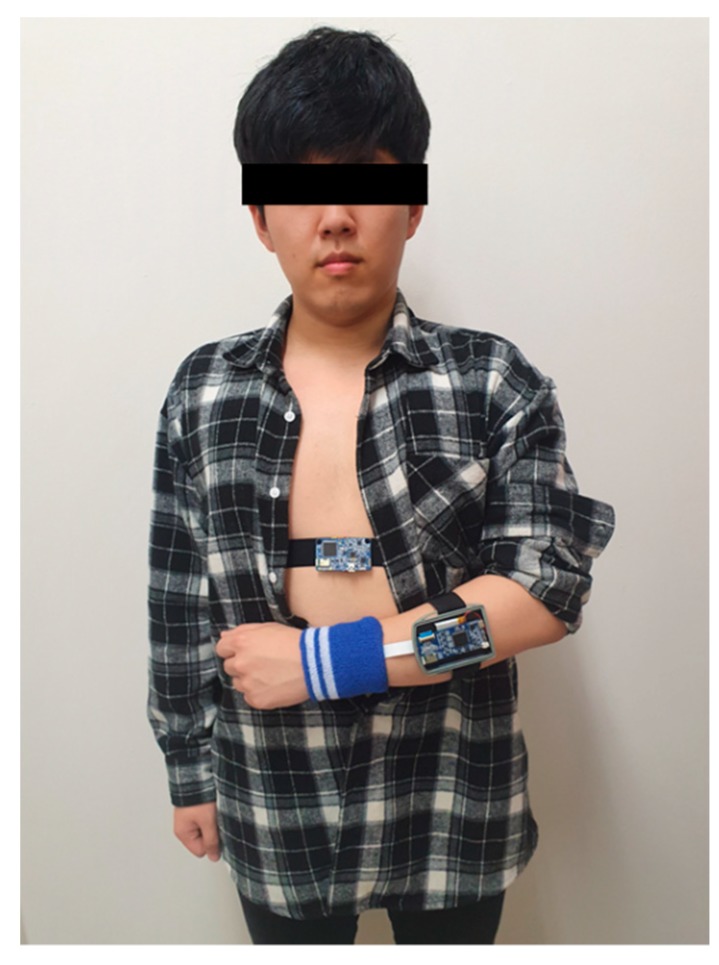
The picture of the subject being equipped with an ECG and multi-channel PPG measurement system.

PPG and ECG were measured in walking, fast walking, and running environments that may occur in everyday life. The developed multi-channel PPG measurement system was worn on the subject’s wrist to measure 12-channel PPG and acceleration signals. The ECG signal to be used as a reference for the heart rate was obtained at a sampling rate of 300 Hz using the developed prototypes of ECG acquisition systems [23] based on ADS1298 (Texas Instruments, Dallas, TX, USA) and the chest strap (Polar Pro Strap, Polar Electro, Ltd., Kempele, Finland). In order to minimize the time delay difference between the multi-channel PPG and ECG systems, two systems can simultaneously perform Analog-to-Digital Conversion (ADC) and transmit measured data based on trigger signals transmitted from the in-house program. Figure 3 shows a photograph of a subject wearing an ECG and multi-channel PPG measurement system. The ECG system for measuring the signal to be used as a reference for HR is mounted on the chest strap, and the main system of the multi-channel PPG measuring system is fixed with a stretchable band on the arm. The sensor module is also fixed with a wrist support band.

Experimental protocols consisted of resting, walking, fast walking, and running sessions. The measured signals were normalized using PPG measured during the first 1-min resting session. In order to minimize the effect between sessions, each session had a 1-min rest period. In the walking (about 1 m/s) session, the subjects walked for 2 min at the typical pace of their daily activities. Next, 2 min of fast walking (about 1.8 m/s) and 2 min of running (about 2.2 m/s) were performed. The experiment was carried out by the subject reciprocating a distance of 20 m in the corridor of the building. Subjects were allowed to move along both sides close to the wall instead of the center of the corridor for a smooth turn of the subject at both turning points. In order to maintain the subject’s speed at each session, the observer induced the subject to reach a fixed time at the start of the round trip. By conducting this protocol two times for each subject, a total of 16 datasets were obtained (one additional dataset whose ECG was damaged by the lead fault of the electrode was excluded). The number of beats for walking, fast walking, and running were 2757, 2913, and 3563, respectively, for a total of 9233 beats. 

### 2.3. Motion Artifact Reduction Algorithm Based on Multi-Sensors

The penetration depth of radiation in human skin is known to increase with increasing wavelengths [24]. When measuring PPG in different directions and locations on the wrist, different signals are detected for the same movement due to the diversity of the directions and distribution of blood vessels in the skin of the wrist. Therefore, in the presence of motion artifacts caused by movement, multi-channel PPG signals measured by sensors with multiple wavelengths and in various locations contain information about blood vessel characteristics under the skin and movement in various directions and depths. 

**Figure 4:**
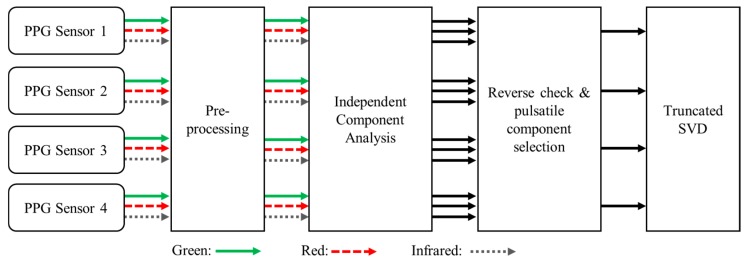
Block diagram of signal flows for each sensor and proposed algorithm.

In this study, we extracted three independent component signals via preprocessing and independent component analysis for three channels of PPG signals with three different wavelengths (green: solid line arrow; red: dashed line arrow; infrared: dotted line arrow) for each of the four sensors, as shown in Figure 4. Among these signals, the signal with the component pulsating the most was selected, and a reconstructed PPG signal was obtained by applying the truncated SVD to a total of four pulsating component signals selected one by one.

**Figure 5:**
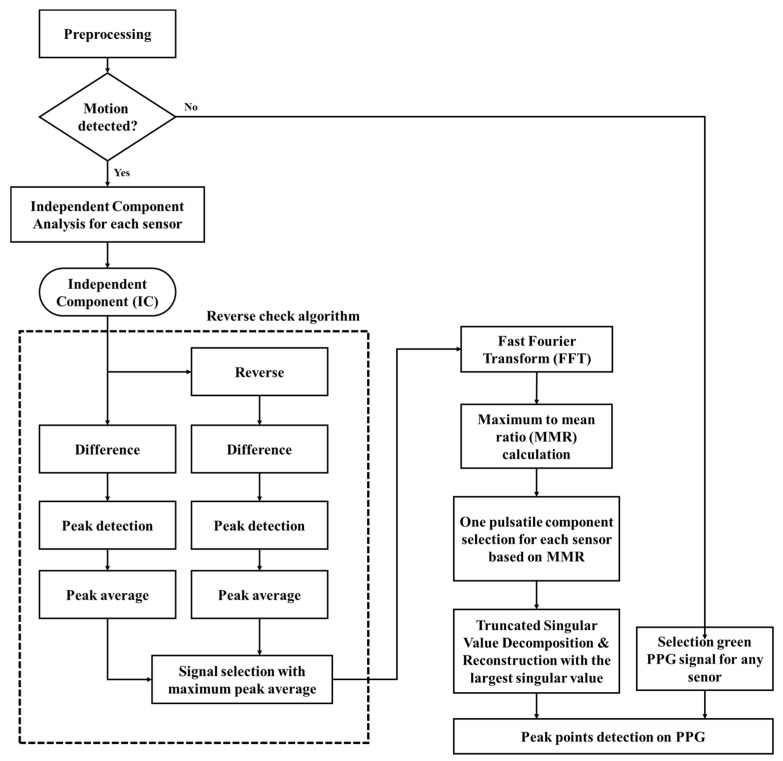
Flowchart of the proposed multi-sensor-based motion artifact reduction algorithm. (MMR is defined as Maximum to mean ratio).

Figure 5 shows the detailed proposed algorithm. The raw signals measured by the sensors contained various noise components such as power line interference, baseline drift, and ambient noise. In order to remove or reduce these noises, digital filtering was performed using a 3-order Butterworth band pass filter with cutoff frequencies of 0.5 and 5 Hz.

In little or no motion environments, all channel signals can be measured with high quality. Therefore, heart rate can only be obtained by detecting peak points in PPG signals without any special processing. In this case, we used a signal with a green wavelength that is known to be measured well on the wrist. To quantify the motion, we used the three-axis accelerometer of the IMU mounted on the sensor module. High-pass filtering was performed to remove the gravity component from the acceleration, and the vector sum ($acc_{sum}$) was calculated for three axes of the gravity-free accelerometer, as shown in Equation (1). The threshold of the presence or absence of motion was set to the vector sum value to distinguish resting and walking.

(1) $$acc_{sum}=\sqrt{(acc_{x}^{2}+acc_{y}^{2}+acc_{z}^{2}})$$

In the presence of movement, PPG signals are a mixture of pulsation and motion artifact components. In this multi-PPG system, the PPG measured by sensors facing different directions showed the change of blood volume of various depths at each location, so the pulsatile component of each sensor can be extracted through ICA algorithms. However, because the output components of the ICA appear in random order and the signals can be reversed, a reverse-check and pulsatile component selection algorithm is required. The reverse-check algorithm (shown as a dotted box in Figure 5) performs differential and peak detection on each independent component (IC), calculates the average of the peak values, and applies the same process to the inverted signal. Due to the morphological characteristics of the PPG, the IC signal with the largest average peak value among the inverted and non-inverted signals becomes the correct PPG. Therefore, the IC with a large average value is the output. 

The number of outputs of the ICA is the same as the number of input channels, and the output signals are randomly output regardless of the order of the input signals. The pulsation component is periodic because it is a change of blood volume that occurs with every beat of the heart. Therefore, by analyzing the periodicity for each IC signal, it is possible to select an IC with a pulsation component. We applied fast Fourier transform (FFT) to IC signals of each sensor module and selected one pulsatile component based on the maximum to mean ratio (MMR) in power spectral density.

As shown in Figure 4, the four components selected based on the MMR were pulsatile components measured at different locations. The truncated SVD [25] was applied to reduce the motion artifacts remaining on the pulsatile components extracted from four sensor modules with different directions, and to obtain the PPG signal that best reflected the change in blood volume. By applying the truncated SVD, the PPG is reflected in the largest singular value. Therefore, reconstruction using only this singular value yields a pure PPG signal with motion artifacts removed. 

**Figure 6:**
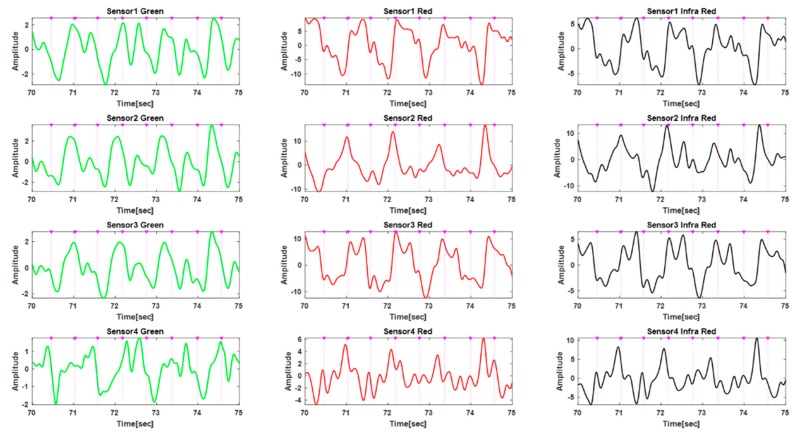
Twelve-channel PPG signals measured while running at about 8 km/h.

**Figure 7:**
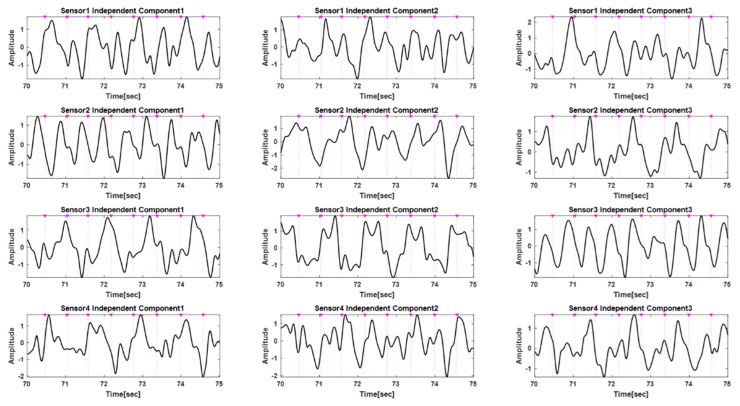
ICA components of 12-channel PPG signals.

**Figure 8:**
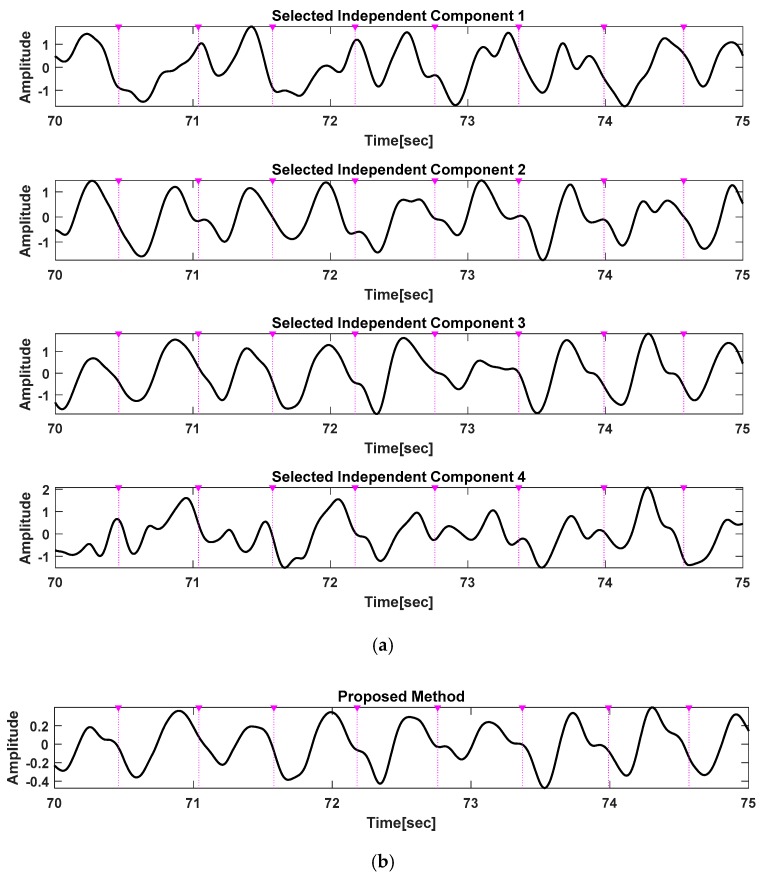
Selected independent components and motion artifact reduced PPG signals. (**a**) Selected ICs applying the reverse-check and pulsatile component selection algorithm, (**b**) reconstructed PPG signal based on the truncated SVD.

Figure 6, Figure 7 and Figure 8 show an example of applying the proposed motion artifact reduction algorithm to a subject’s data. Figure 6 shows the 12-channel PPG signals measured in the running state most affected by motion artifacts. The *x*-axis represents the time elapsed after 70 s, including 1 min of resting and 10 s of running.

Figure 7 and Figure 8 show the results of applying the algorithm to each step shown in Figure 5. Figure 7 shows the 12 ICs after passing the ICA. Figure 8a shows four ICs selected through pulsatile component selection for each sensor module, and Figure 8b shows the results of applying the truncated SVD. 

Figure 6 shows a 12-channel PPG signal measured from sensors placed in four directions while running at about 8 km/h. The four rows represent signals measured by the four sensor modules, and each column is a frequency-specific (green, red, and infrared) signal that can reflect information of the same depth. It can be seen that all channels are contaminated by motion artifacts. The R-peak time points extracted from the ECG are marked with the symbol (▼), and purple dotted lines represent the heart rate. The Pan–Tompkins algorithm was used to detect R-peaks from ECG data [26]. In this paper, all algorithms (including the motion artifact reduction algorithm) were implemented in Matlab 2019 (MathWorks Inc, Natick, MA, USA).

Figure 7 shows the 12 independent components (ICs) applying ICA to the 12-channel input PPG signals shown in Figure 6. This represents the same number of ICs as the number of input signals and includes pulsatile, motion artifacts, and other noise components for the signals acquired from each sensor. Each component appears in random order and some may appear as inverted signals. Each sensor module has three ICs for PPG signals from three wavelengths, which are the signals shown in each row of Figure 7. 

In Figure 7, the most periodic signals for the IC signals and their inverted signals for each sensor module are IC1, IC1, and IC3 for the sensor modules 1, 2, and 3, respectively; the signals for sensor 4 are unclear. The results of applying the reverse-check and pulsatile component selection algorithm to select these pulsatile components are shown in Figure 8a. One pulsatile signal was selected for each sensor. In this example, the signals selected for each of the sensor modules 1, 2, and 3 are the reversed signals of IC1 and the reversed signal of IC1 and IC2. After applying the truncated SVD to the four selected ICs, reconstruction using only the largest singular value yielded a pure PPG signal with motion artifacts reduced, as shown in Figure 8b. 

## 3. Experimental Results

In order to evaluate the proposed algorithm, the motion artifact reduction algorithm was applied to the signal measured by the multi-channel PPG measurement system, and the indexes for evaluating the performance of the algorithm were calculated by comparing the ECG with the reference signal. Sensitivity (Se) as Equation (2), positive predictive value (PPV) as Equation (3), and failed detection rate (FDR) as Equation (4) were used as performance indexes [27]. In these equations, TP (True Positive) is the number of peaks detected, FN (False Negative) is the number of peaks non-detected, and FP (False Positive) is the number of artifacts or noise classified as peaks. Algorithms based on the best signal selection [15], ICA [28], or SVD [25] were compared with the proposed method and evaluated using performance indexes.

(2) $$\mathrm{Se}=\;\frac{TP}{TP+FN}\times 100\%$$

(3) $$\mathrm{PPV}=\frac{TP}{TP+FP}\times 100\%$$

(4) $$\mathrm{FDR}=\frac{FP}{TP}\times 100\%$$

**Figure 9:**
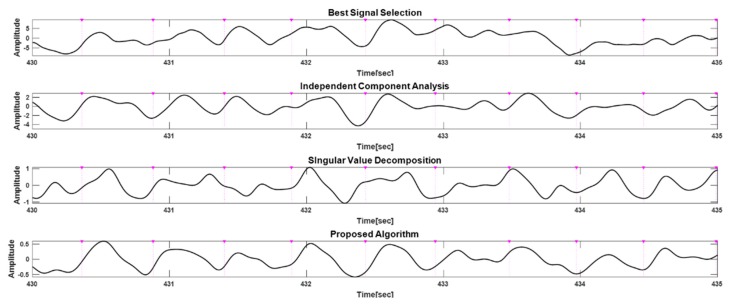
Example of applying the proposed method and algorithms based on best signal selection, ICA, and SVD during running at about 8 km/h.

Figure 9 shows the results of applying the proposed method and algorithms based on the best signal selection, ICA, or SVD to the measured data at about 8 km/h. The R-peak time points extracted from the ECG as a reference for the heartbeat are indicated by the (▼) symbol and purple dotted lines. Compared with other methods, the proposed algorithm is clearest in terms of the heart rate extraction of PPG signals and had a low FDR. 

Table 1 shows Se, PPV, and FDR as performance indexes for peak detection on 12-channel data measured during walking, fast walking, and running. As the intensity of the motion increases, Se decreases and PPV changes less. From the results, it can be seen that as the motion artifacts became more severe, the false negative (FN) for beat detection increased a lot while the false positive (FP) tended to fall slightly. In terms of wavelength, the green wavelength channels Ch1, Ch4, Ch7, and Ch10 had high sensitivity.

Table 2 shows the performance parameters Se, PPV, and FDR from algorithms based on the best selection method, SVD, ICA, and proposed method for the data measured in walking (2757 beats), fast walking (2913 beats), and running (3563 beats). As the movement increased with walking, fast walking, and running, the detection rate of heart beats decreased. The performance of the proposed method for walking was 99%, 99.55%, and 0.45% for Se, PPV, and FDR, respectively. The performance of the proposed method was higher than that of the other algorithms, and the SVD-based algorithm had the lowest accuracy. For the fast walking and running conditions, the proposed method showed the best performance, as well as for these two conditions with a lot of motion. 

## 4. Discussion

ECG is a representative signal for calculating heart rate that measures the bio-potential generated by electrical signals that control the expansion and contraction of the heart. Another signal is that of PPG, a light-based technology to sense volumetric changes in blood in peripheral circulation as controlled by the heart’s pumping action. ECG produces an electrical signal that is robust in the presence of motion artifacts and has the advantage of stably extracting heartbeats even compromised by motion. However, ECG signals are obtained by measuring a weak electrical potential difference between two points, and thus cannot be measured on a single arm. In the case of using both hands with more electrical potential difference, the user must intentionally make contact with the electrode. In contrast, PPG has an advantage over ECG in terms of user convenience and wearability, as it can take measurements in any location with a high concentration of blood vessels.

Over the past decade, there HR monitors and wearable fitness equipment have been made commercially available. Many people use HR monitors to inform their training and to access aerobic fitness. 

In the clinical field, physicians and trainers often refer to physiological and behavioral data such as energy consumption, step counts, sleep/wake information, and HR obtained from patients’ wearable devices. Energy consumption is calculated using HR and accelerometer motion information. If accurate HR information is provided, more accurate energy consumption can be estimated. Accurate HR monitoring is an essential component of a systematic exercise prescription because a target HR is set to guide patient-specific exercise intensity. 

As another field of the clinical application of HR, heart rate variability (HRV) is widely used to investigate the state of autonomic nervous systems and related diseases. HRV is calculated using beat-by-beat HRs, and accurate HR detection is required because HR errors lead to false ANS (Autonomic nervous system) analysis.

Currently, many devices are being used to monitor heart rate in the form of bands or watches (e.g., Apple watch, Fitbit, and Samsung Gear), and these PPG devices have the advantages of being lightweight, portable, and easy to use. However, the PPG signal is very vulnerable to motion artifacts. It is well known that the depth of penetration of light into human skin increases with decreasing wavelength [29]. Therefore, the wavelength of the LED and the direction of the PPG sensor module are important factors for analyzing the influence of motion artifacts in the PPG signal. In this study, we developed a 12-channel PPG measurement system with up, down, left, and right directions using three wavelengths (green, red, and infrared) for each direction. In addition, we proposed a multi-channel PPG motion artifact reduction algorithm based on independent component analysis and truncated singular value decomposition. In this study, PPG signals were measured at multiple skin penetration depths according to LED wavelength. In addition, PPG signals for each position and direction of blood vessels in the skin were measured through sensor modules with up, down, left and right directions. In order to apply independent component analysis to multi-channel PPG signals, signal inversion and pulsation component detection ability were confirmed by comparing MMR in the power spectral density through FFT. Furthermore, truncated singular value decomposition was applied to the detected pulsating components to reduce motion artifacts. When the proposed algorithm was applied to the signal measured during running using the developed system, a sensitivity of 82.49%, a positive predictive value of 99.83%, and a false detection rate of 0.17% were obtained. These results are due to the motion artifact reduction algorithm applied to the multi-channel PPG measurement system using various wavelengths and directions of the sensor modules. As the wavelengths increased, the change in blood volume at a deeper depth could be measured. Therefore, each PPG has a different amount of information, and when applied to the ICA algorithm, a pulsatile component can be obtained. In addition, the pulsatile components acquired from the sensor modules in different directions are represented as a single PPG signal that shows the change in blood volume without being affected by motion through the truncated SVD.

The proposed algorithm extracts the independent component signals from PPG signals with different wavelengths measured for each sensor module, as shown in Figure 4. In addition, in order to compare the effect of the wavelength and the direction of the sensor, we applied the proposed algorithm for PPG signals with the same wavelength in each senor module. This approach did not provide clear results as compared with the use of ICA-based algorithms for each sensor module. This means that signals measured by LEDs of the same wavelength in each sensor are measured through different positions and paths of light, so that information on motion artifacts is inconsistent when applying independent component analysis. Therefore, applying independent component analysis to the signals measured by the same sensor module rather than the same wavelength signals can increase the motion artifact reduction performance.

The limitation of this study is that the protocols for PPG measurement were limited to walking, fast walking, and running; therefore, more detailed protocols need to be set up to remove the noise caused by various kinds of movements in real life. Additional experiments and research are needed to develop real-world applications. As further work, we plan to quantitatively analyze the effects of motion artifacts on PPG signals at various speeds on the treadmill. In addition, long-term measurements are required to analyze the various features in the signal measured during physical activity. We will try to diversify the protocols and measure data in various environments through long-term experiments.

The proposed method is effective for monitoring heart rate. Furthermore, if the beat-to-beat interval can be obtained precisely like the R-peak of ECG in the presence of motion artifacts, it can be applied to various fields, such as emotion or stress, by analyzing the autonomic nervous system based on pulse rate variability.

## Figures and Tables

**Table 1:** Comparison of the results of Se, PPV, and FDR for the 12-channel signals acquired during walking, fast walking, and running conditions.

Conditions	Performance Parameters	Channels					
		Ch1	Ch2	Ch3	Ch4	Ch5	Ch6
**Walking**	Se (%)	93.66	91.14	92.22	97.07	88.33	91.27
	PPV (%)	97.63	92.19	94.03	99.10	92.77	93.99
	FDR (%)	2.43	8.47	6.35	0.91	7.79	6.39
		Ch7	Ch8	Ch9	Ch10	Ch11	Ch12
	Se (%)	96.59	88.46	90.36	96.48	90.39	92.10
	PPV (%)	99.47	92.96	95.96	98.30	92.68	94.80
	FDR (%)	0.53	7.57	4.21	1.73	7.90	5.49
**Fast walking**		Ch1	Ch2	Ch3	Ch4	Ch5	Ch6
	Se (%)	84.17	80.47	82.80	93.69	77.07	79.73
	PPV (%)	98.23	94.10	95.70	99.08	94.07	95.48
	FDR (%)	1.80	6.27	4.49	0.93	6.30	4.73
		Ch7	Ch8	Ch9	Ch10	Ch11	Ch12
	Se (%)	91.03	78.95	82.74	90.05	80.50	84.64
	PPV (%)	99.43	93.60	95.21	98.58	94.29	95.04
	FDR (%)	0.57	6.84	5.03	1.44	6.06	5.22
**Running**		Ch1	Ch2	Ch3	Ch4	Ch5	Ch6
	Se (%)	72.48	67.67	68.68	76.53	66.54	68.21
	PPV (%)	99.42	99.12	99.41	99.60	99.43	99.17
	FDR (%)	0.58	0.89	0.59	0.40	0.57	0.84
		Ch7	Ch8	Ch9	Ch10	Ch11	Ch12
	Se (%)	77.28	67.53	69.36	76.60	66.14	66.94
	PPV (%)	99.57	99.05	99.29	99.69	98.75	99.04
	FDR (%)	0.43	0.96	0.72	0.31	1.27	0.97

**Table 2:** Comparison of the results of Se, PPV, and FDR from algorithms based on the best signal selection method, SVD, ICA, or the proposed method for signals acquired under the walking, fast walking, and running conditions.

Conditions	Performance	Best Signal Selection	SVD	ICA	Proposed
**Walking**	Se (%)	97.07	91.30	98.65	99.00
	PPV (%)	99.10	92.89	99.03	99.55
	FDR (%)	0.91	7.65	0.98	0.45
**Fast walking**	Se (%)	93.69	82.33	95.42	96.28
	PPV (%)	99.08	93.71	99.06	99.24
	FDR (%)	0.93	6.71	0.95	0.77
**Running**	Se (%)	77.28	68.00	79.98	82.49
	PPV (%)	99.57	99.09	99.81	99.83
	FDR (%)	0.43	0.92	0.19	0.17
